# Dynamic holographic display with addressable on-chip metasurface network based on lithium niobate photonics

**DOI:** 10.1038/s41377-025-02014-6

**Published:** 2025-09-18

**Authors:** Jitao Ji, Zhilin Ye, Zhizhang Wang, Jiacheng Sun, Xueyun Li, Jian Li, Junyi Wang, Bin Fang, Zihan Gao, Shanshan Hu, Shining Zhu, Tao Li

**Affiliations:** National Laboratory of Solid State Microstructures, Key Laboratory of Intelligent Optical Sensing and Manipulations, Jiangsu Key Laboratory of Artificial Functional Materials, College of Engineering and Applied Sciences, Nanjing University, Nanjing, 210093 China

**Keywords:** Nanophotonics and plasmonics, Nanophotonics and plasmonics

## Abstract

On-chip metasurfaces have been exploited to manipulate guided waves into free space with desired wavefronts and bridge the gap between guided modes and free-space optical fields. However, existing on-chip metasurfaces for guided wave radiation typically lack dynamic tunability and high-capacity multiplexing for their practical applications. Here, we present a dynamic waveguide-based holographic display enabled by on-chip metasurface network on lithium niobate on insulator platform. Based on geometric phase and detour phase, an on-chip multiplexed metasurface network implemented on a 2 × 2 waveguide crossing array is incorporated with a two-stage lithium niobate (LN) switch to construct an addressable framework. Benefiting from the multiplexing capability of on-chip metasurface and the fast electro-optical response of LN modulators, guided wave radiation in the form of eight holographic images is tunable and addressable at high speeds. This work exemplifies a scalable approach for dynamic manipulation of guided signals and paves the way towards holographic displays, high-capacity optical communications and integrated photonic information processing.

## Introduction

Endowed with powerful optical field manipulation capabilities at subwavelength scale, metasurfaces have showcased exceptional promise across diverse optical applications in the field of holographic display^1^, augmented reality^2^, optical imaging^3^, quantum optics^4^, etc^5,6^. Apart from the significant advances in free space, metasurfaces have been recently incorporated with optical waveguides to establish building blocks of photonic integrated circuits (PICs) with expanded functionalities and improved performance^7–9^. Such on-chip metasurfaces enable flexible manipulation of guided signals and redefine numerous photonic integrated devices^10–18^. In particular, guided wave-driven metasurfaces which radiate guided waves into free space with desired beam forms, could serve as an upgraded version of conventional out-coupler^19–27^. Owing to the on-chip optical propagation scheme, such guided wave radiation is featured with compact device size and no zero-order diffraction, which would contribute to the integration and high performance of optical display technologies.

To date, many efforts in on-chip metasurfaces have been invested into multi-channel guided wave radiation to achieve enhanced multiplexing capability through joint phase modulation^28,29^, z-plane spatial multiplexing strategies^30,31^ and other mechanisms^32,33^. Nevertheless, there is still plenty of room to further increase the number of multiplexed channels for practical scenarios. On the other hand, how to realize dynamic modulation capability has been a long-standing problem for metasurface devices. Despite that liquid crystal^34^ and liquid-immersive strategy^35,36^ have been attempted to acquire switchable modulation, they exhibit low response speeds. Moreover, most of previous tunable metasurfaces are designed in global phase modulation, which lacks local or pixelated modulation functionality. Therefore, achieving large-capacity, pixel-level and fast-speed tunability poses considerable challenges to existing metasurface devices (see Supplementary Information Note 1 for the metrics and performance of the existing metasurfaces).

Due to the on-chip arrangement scheme, metasurfaces allow spatial expansion and could contribute to the on-chip metasurface network (OCMN) for multi-channel multiplexing^20^. Moreover, waveguide crossing array (WCA) has demonstrated its ability to provide a reliable configuration for addressable signal processing^37–39^. Accordingly, combining OCMN with WCA is expected to present a solution to large-capacity and pixelated addressable manipulation. Furthermore, with the rapid development of lithium niobate on insulator (LNOI), lithium niobate (LN) has become one of the promising platforms to construct next-generation PICs^40–45^. With the evolution of high-performance LN electro-optical modulators^46–48^, LN Mach-Zehnder interferometers (MZI) have recently been proposed to integrate with the on-chip metasurface and have exhibited reconfigurable wavefront shaping of guided wave radiation with gigahertz-rate speed^49^, which empowers efficient and ultra-fast dynamic management of guided waves.

In this work, we demonstrate an OCMN facilitated by a 2 × 2 LN WCA for addressable holographic display on LNOI platform. Through joint modulation of geometric phase and detour phase, polarization-multiplexed and illumination direction-multiplexed on-chip metasurface has been optimized as a basic addressing unit. On this basis, through two-dimensional spatial expansion, the OCMN is implemented on a 2 × 2 LN WCA with four input ports to demonstrate static tuning functionality. Furthermore, such OCMN is connected with a two-stage LN MZI switch consisting of three LN modulators to perform dynamic switching of holographic display. As a proof-of-concept demonstration, eight holographic images are experimentally addressed with a nanosecond-level response speed according to the applied voltages and analyzed polarization states. This on-chip scheme is scalable and compatible with other photonic integrated devices, which may open up a new perspective for display technology, free-space communications and information encoding.

## Results

### Concept of addressable on-chip metasurface network

**Fig. 1:**
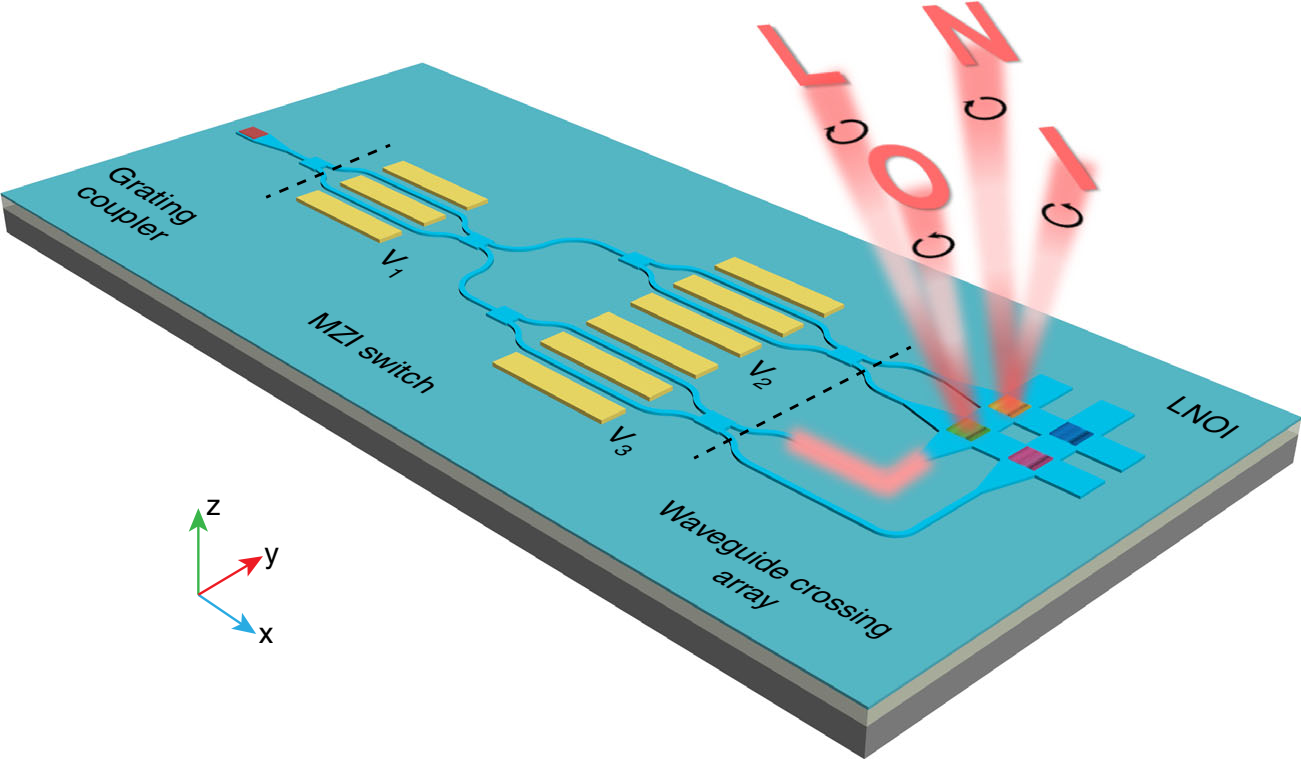
Schematic illustration of addressable OCMN based on grating coupler, LN MZI switches and a 2×2 WCA on LNOI platform

As the conceptual diagram shows in Fig. 1, the proposed addressable OCMN could be understood as three procedures, namely grating coupling, optical routing and guided wave radiation. To be specific, the guided mode is excited by grating coupler from free-space incidences and propagates in a single-mode ridge LN waveguide. The guided mode is subsequently split into a first-stage LN modulator through a 1 × 2 multi-mode interference (MMI) coupler, followed by two second-stage LN modulators which route the guided mode into four output ports via two 2 × 2 MMI couplers. By virtue of electro-optical modulation, the guided mode could be switched into a specific output port at high speeds and routed into one of the four input ports in succeeding 2 × 2 LN WCA via tapered adiabatic waveguides. Once the guided mode propagating within WCA encounters on-chip metasurfaces which could be regarded as an addressing unit in OCMN, it will be decoupled into free space and modulated into desired optical field distributions. Owing to the weak interaction process, the guided mode could be successively extracted by on-chip metasurfaces (e.g. the green and yellow ones in Fig. 1) positioned along the propagation direction of the guided mode. All the on-chip metasurfaces are based on geometric phase and detour phase modulation mechanisms, each of which possesses four multiplexed channels related to the illumination direction and output polarization states. Hence, through controlling the input ports of LN WCA via LN MZI switch, different guided wave radiation in the form of holographic images could be dynamically tunable. This configuration not only offers sufficient number of multiplexed channels enabled by OCMN, but also develops a dynamic scheme of holographic display and exploits the prototype of scalable addressing capability on LNOI platform.

### Design of on-chip metasurface

**Fig. 2:**
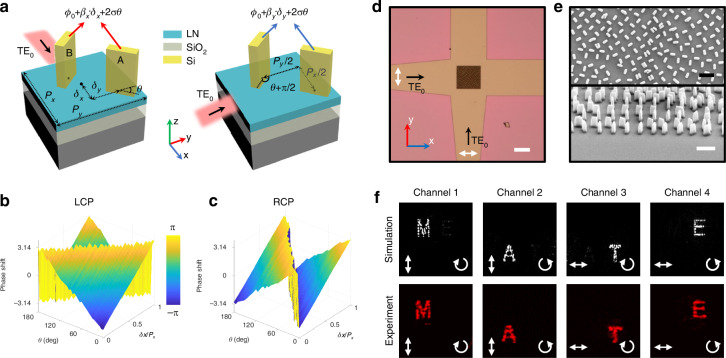
Design of on-chip metasurface. **a** Principle of phase modulation of diatomic structure based on geometric phase and detour phase. Simulated phase shift map of (**b**) left circularly polarized (LCP) and (**c**) right circularly polarized (RCP) output channel as a function of rotation angle *θ* and displacement *δ*_*x*_. **d** Optical microscope image of the fabricated on-chip metasurface on top of LN waveguide crossing. Scale bar: 50 μm. **e** Scanning electron microscope images of the on-chip metasurface. Scale bars: 1 μm. **f** Simulated and measured four-channel holographic images under TE_0_ mode illuminations along x and y directions along with LCP and RCP analyzers. The double-headed arrows correspond to the polarization state of the input TE_0_ mode and the circular arrows represent the polarization state of the analyzer

To construct OCMN, the on-chip multiplexed metasurfaces are designed as an addressing unit and made up of amorphous silicon nanopillars (with 360 nm length, 120 nm width and 600 nm height) on an x-cut LNOI chip. As depicted in Fig. 2a, implemented on top of a 600 nm-thick slab LN waveguide, the on-chip metasurfaces are featured with a diatomic structure with periods of *P*_*x*_ and *P*_*y*_ (corresponding to the effective wavelength λ_*effx*_ = 790 nm and λ_*effy*_ = 760 nm of TE_0_ modes) along x and y directions. Such a meta-diatom comprises two identical but orthogonally arranged nanopillars (A and B) with x and y displacements of *P*_*x*_/2 and *P*_*y*_/2. Here, geometric phase is incorporated with detour phase to provide polarization-multiplexed and illumination direction-multiplexed guided wave radiation. The phase profiles of the extracted wave can be expressed as (derived in Supplementary Information Note 2)

1 $$\begin{array}{l}{\phi }_{xL}={\beta }_{x}\cdot {\delta }_{x}+2\theta \\ {\phi }_{xR}={\beta }_{x}\cdot {\delta }_{x}-2\theta \\ {\phi }_{yL}={\beta }_{y}\cdot {\delta }_{y}+2\theta \\ {\phi }_{yR}={\beta }_{y}\cdot {\delta }_{y}-2\theta \end{array}$$

where ${\phi }_{xL}$, ${\phi }_{xR}$, ${\phi }_{yL}$ and ${\phi }_{yR}$ are the phase responses of left and right circularly polarized (LCP and RCP) radiation under TE_0_ guided wave illuminations along x and y directions. Here, *β*_*x*_ and *β*_*y*_ correspond to the propagation constants of TE_0_ mode propagating along x and y directions. *δ*_*x*_ and *δ*_*y*_ are the displacements of nanopillar A within the periods *P*_*x*_ and *P*_*y*_ while *θ* represents the rotation angle of nanopillar A. Due to the diatomic arrangement, the unmodulated part of the extracted wave is eliminated by destructive interference and the signal-to-noise ratio (SNR) could be effectively improved (see Fig. S2). To demonstrate the feasibility of joint modulation by geometric phase and detour phase, numerical simulations were performed to investigate the phase response induced by meta-diatoms (see Methods). For simplicity, only the case under guided wave illumination along the x direction is considered. As illustrated in Fig. 2b and c, the phase shifts of the extracted LCP (RCP) wave exhibit a linear relationship with rotation angle *θ* by a factor of 2 (-2) and increases as a function of *δ*_*x*_/*P*_*x*_. These results indicate that the phase shifts caused by geometric phase and detour phase can be independently modulated and linearly superimposed, contributing to the four multiplexed channels expressed in Eq. (1). Nevertheless, such four phase channels are restricted by the relation of ${\phi }_{xL}-{\phi }_{xR}={\phi }_{yL}-{\phi }_{yR}$, since only three design variables *δ*_*x*_, *δ*_*y*_ and *θ* exist. To solve this problem, a modified Gerchberg-Saxton (G-S) algorithm was employed to optimize the phase distributions of four multiplexed channels for encoding four holographic images with three design variables (see Supplementary Information Note 3). In addition, as the TE_0_ guided wave illumination experiences an adiabatic evolution from a single-mode waveguide to a slab region with on-chip metasurface, a spherical incident wavefront forms as an initial phase for guided wave radiation. Therefore, a phase correction process has been performed on the phase distributions of the metasurface along both x and y directions to compensate the bent incident wavefront (see Supplementary Information Note 4). After determining the phase distributions of four channels, the three variables *δ*_*x*_, *δ*_*y*_ and *θ* could be ascertained for on-chip multiplexed metasurfaces according to Eq. (1).

As an experimental demonstration, we prepared the on-chip metasurface on a LN waveguide crossing with an etch depth of 300 nm through the fabrication process detailed in Methods. Figures 2d and e present the optical microscope image and scanning electron microscope image of the fabricated on-chip metasurface with 100×100 pixels, respectively. The performance is experimentally investigated through a custom-built measurement setup illustrated in Methods. As shown in Fig. 2f, due to the spin-decoupled properties of on-chip metasurface, the projected holographic images under the illuminations along the x direction can be flexibly converted from “M” to “A” by tuning the state of the polarization analyzer from LCP to RCP. Through switching the illuminations along the y direction, another two holographic images are addressed and emerge as “T” and “E” with LCP and RCP states. This illumination direction-multiplexed feature lays the foundation for addressable OCMN. Notably, the observed four images are free from zero-order diffraction and exhibit negligible crosstalk to other channels (see Supplementary Information Note 5 for analysis), which is highly consistent with simulation results. In experiments, the extraction efficiency of the on-chip metasurface, which is defined as the ratio of the power of the extracted guided wave radiation to the power of input guided wave, is measured to be ~6%. In addition, benefiting from the broadband characteristics of both geometric phase and detour phase, such on-chip metasurface can operate over a broadband spectrum ranging from 1500 nm to 1590 nm (see Supplementary Information Note 6 for details). Apart from holographic images, this multiplexed functionality could also be extended into other forms and the generation of multiplexed orbital angular momentum beams is exemplified in Supplementary Information Note 7.

### Demonstration of holographic display through on-chip metasurface network

**Fig. 3:**
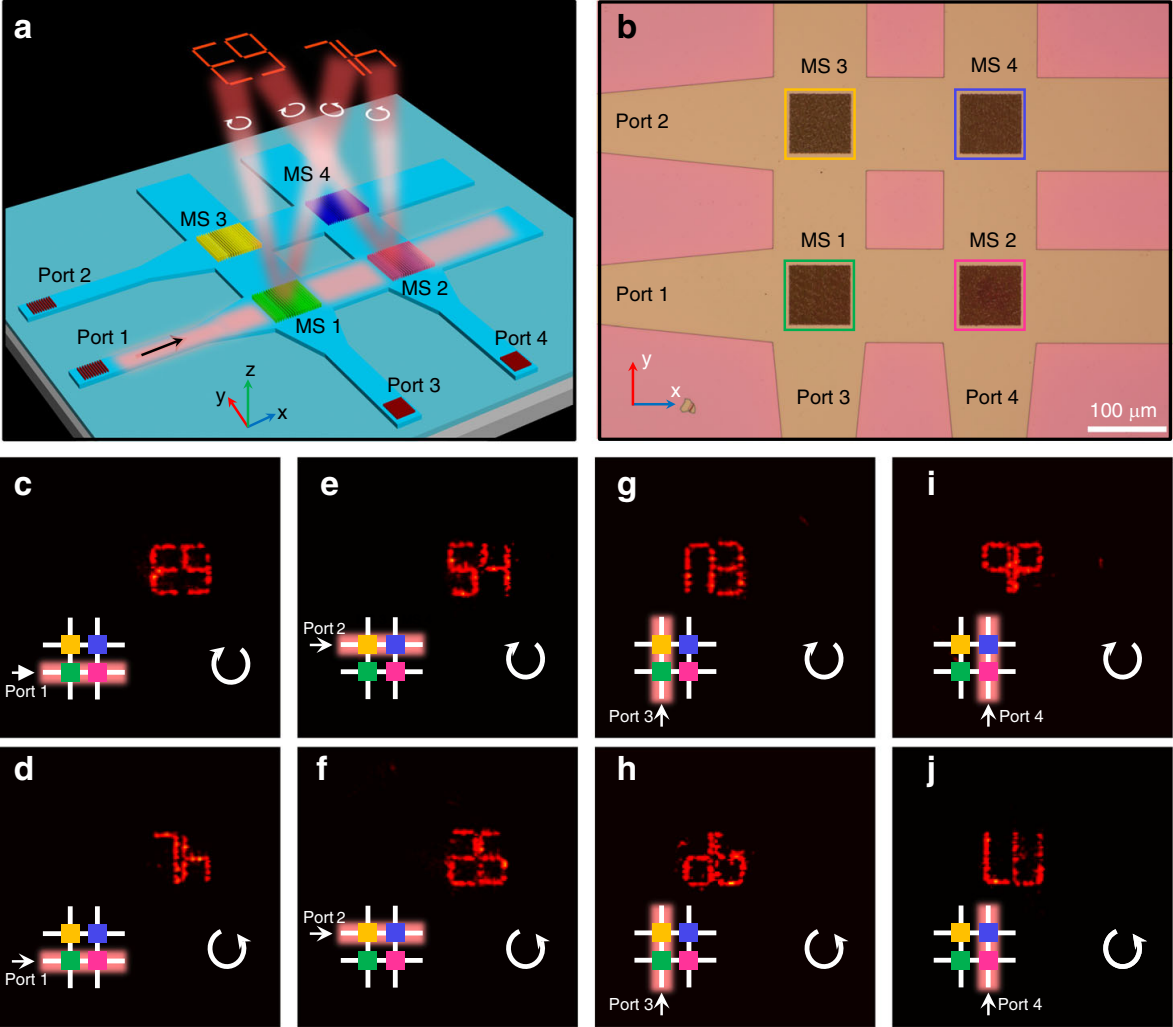
Demonstration of switchable holographic display enabled by OCMN. **a** Conceptual diagram of OCMN activated by LN WCA with four metasurfaces (MS 1-4) and four input ports (Port 1-4). **b** Optical microscope image of the fabricated OCMN where green, magenta, yellow and blue boxes outline the locations of MS 1-4. Scale bar: 100 μm. Measured far-field holographic images generated by the OCMN when TE_0_ mode is illuminated from **c**, **d** Port 1, **e**, **f** Port 2, **g**, **h** Port 3, and **i**, **j** Port 4, respectively. The diagrams in the left corners indicate the input port and the corresponding excited metasurfaces in OCMN while the circular arrows in the right corners represent the analyzed circular polarization states

Since guided wave radiation by on-chip metasurface is a weak interaction process^20,29^, it is feasible to extract guided waves successively with nearly uniform intensity along the propagation direction. As a consequence, on-chip metasurface can be expanded into OCMN along two-dimensional space (i.e., cascaded along both x and y directions) through WCA scheme, as depicted in Fig. 3a. Under this circumstance, the input ports of WCA can be regarded as encoding addresses. Different holographic images can thereby be addressed by switching the input ports to activate specific cells in on-chip metasurface network. For demonstration, a 2 × 2 OCMN indicated in Fig. 3b containing four on-chip metasurfaces (MS 1-4) and four input ports (Port 1-4) is prepared. Then, incident light with λ = 1550 nm is coupled into four input ports individually and the corresponding generated holographic images are recorded. When a guided wave is excited in Port 1 and propagates along x direction, metasurfaces circled by green and magenta boxes are triggered and project holographic images of “E9” and “7 h” in the far field with LCP and RCP analyzers, see Fig. 3c, d. Through switching the input ports from Port 1 to Port 2-4 and tuning the analyzed polarization states, another six holographic letters and numbers can be read out as listed in Fig. 3e–j, validating addressable the capability of OCMN (refer to Supplementary Information Note 8 for designed holographic patterns of MS 1-4). Furthermore, such design is highly scalable and promising to realize large-scale OCMN for optical information memory and encoding.

### Dynamic manipulation based on LN MZI switch

**Fig. 4:**
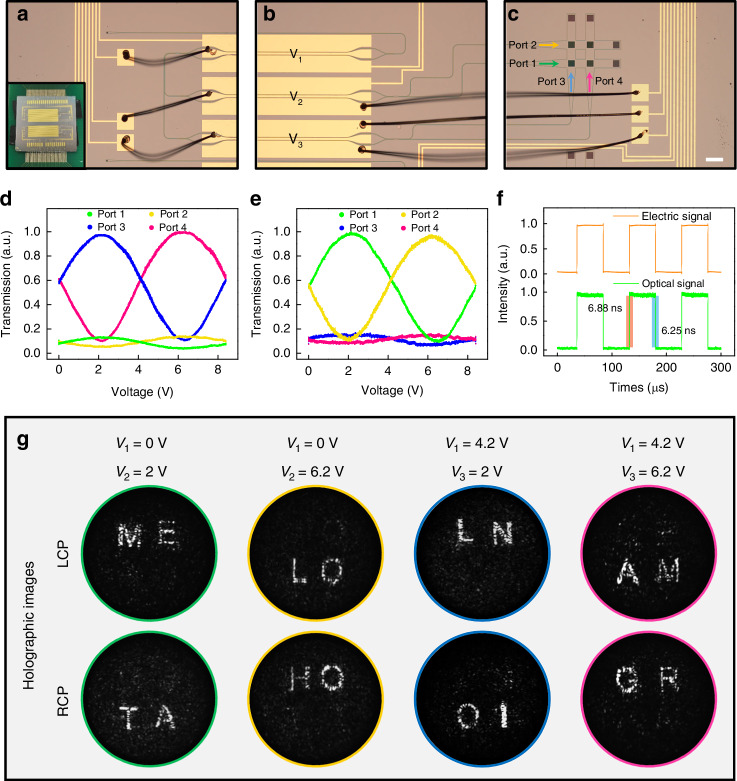
Characterization of dynamic holographic display enabled by LN MZI switch. Images of the fabricated sample composed of (**a**) grating coupler, **b** two-stage LN MZI switch with applied voltages V_1_, V_2_, V_3_, and (**c**) OCMN including four on-chip metasurfaces with colored arrows indicating four input ports. Scale bar: 200 μm. The photograph of the sample is shown in the inset of (**a**). **d** The transmission of four input ports as a function of V_2_ with constant V_1_ of 0 V. **e** The transmission of four input ports as a function of V_3_ with constant V_1_ of 4.2 V. **f** Response of LN modulator after applying a square-wave voltage signal with 10 kHz. **g** Measured holographic images upon switching applied voltages with LCP and RCP analyzers

After verifying the addressable capability in a static level, we proceed to connect such OCMN with LN electro-optical MZI switch composed of four output ports to acquire dynamic holographic display functionality. Figure 4a–c present the fabricated sample including the regions of the grating coupler, a two-stage LN MZI switch and OCMN implemented on a 2 × 2 WCA. The two-stage LN MZI switch consists of three LN modulators, each with the same physical size. To achieve high performance of optical switching, the electrode length and gap of LN modulators have been optimized to be 10 mm and 6.5 μm, respectively, leading to a low half-wave voltage V_π_ of 4.2 V in experiments (see Supplementary Information Note 9 for detailed design of LN devices). As a consequence, the intensity of all four output ports could be assigned as required by tuning the applied voltages V_1_, V_2_ and V_3_ in three modulators. As plotted in Fig. 4d (4e), when V_1_ is fixed at 0 V (4.2 V), the output intensities in Ports 1 and 2 (Ports 3 and 4) show sinusoidal relationship with applied V_2_ (V_3_) while maintaining the intensities in Ports 3 and 4 (Ports 1 and 2) almost unchanged. This result proves the feasibility of optical switching enabled by LN MZI switch. Moreover, we characterize the response time of LN modulators to be ~6.5 ns through applying a 10 kHz square wave voltage, as shown in Fig. 4f, which ensures high-speed modulation. On this basis, dynamic performance is subsequently explored by actuating LN MZI switch to assign “a specific address” to activate the on-chip metasurface network (see Methods for details of experimental setup). As illustrated in Fig. 4g, by switching the applied voltages V_1_, V_2_ and V_3_, the radiated holographic images could be flexibly read out as different letters with LCP and RCP states at 10 Hz switching speed (limited by the frame rate of the camera) with the related video provided in Supplementary Video. For instance, this OCMN displays “ME” and “TA” at V_1_ = 0 V, V_2_ = 2 V while switches to “LO” and “HO” at V_1_ = 0 V, V_2_ = 6.2 V under LCP and RCP analyzers, respectively. Overall, high-speed dynamic holographic display empowered by the LN MZI switch and the addressable metasurface network has been demonstrated, pointing out a promising OCMN framework for large-scale processing capability of on-chip optical signals.

## Discussion

In summary, we propose a fast modulation strategy for dynamic holographic display with improved multiplexing capability on LNOI platform. With joint phase modulation of geometric phase and detour phase, a diatomic multiplexed on-chip metasurface is designed to serve as an addressing unit by virtue of modified a G-S optimization algorithm. As an extension, an OCMN facilitated by 2 × 2 WCA has been established for addressable manipulation between eight holographic images by switching different input ports and polarization analyzers. Furthermore, we employ three LN modulators to construct a two-stage LN MZI switch to control the four illumination ports, thus enabling dynamic tunability with spatially expandable OCMN. Despite the fact that an integrated configuration for dynamic control of free-space optical field radiation has only been demonstrated in the form of holographic display as an example in this work, the proposed OCMN framework driven by LN switch provides a universal and scalable integrated platform for fast dynamic control and large-capacity modulation and enables other attractive functions. We envision that cutting-edge metasurface design methods and algorithm optimization strategies could be additionally introduced into our configuration to simultaneously expand the number of modulation channels and improve the quality of guided wave radiation, thereby promoting more extensive applications like imaging, display, free-space optical interconnects, and optical communications.

## Materials and methods

### Numerical simulations

The finite-difference time-domain (FDTD) method was employed to calculate the phase response of meta-atoms and far-field distributions of the total on-chip metasurface. A 1550 nm mode source was placed at the cross section of the LN waveguide to excite TE_0_ guided mode along x and y directions. Perfectly matched layer (PML) conditions were applied along all the boundaries. The refractive indices of LN, amorphous silicon and silicon oxide used in simulations were obtained from experimental samples. Then, the phase response of meta-atoms and the extracted guided wave by on-chip metasurfaces were collected by point monitor and surface monitor, respectively.

### Device fabrication

The samples were prepared on a commercial x-cut LNOI wafer with a 600 nm LN layer and a 2 μm buried silicon dioxide. A layer of ma-N2405 was first spin-coated on the LN thin film as a mask. The waveguide patterns were subsequently defined through an E-beam lithography (EBL) process and transferred into LN layer with an optimized argon plasma in an ion beam etching (IBE) system. After removing the resist and cleaning the LN sidewall, an α-Si layer with a thickness of 600 nm was deposited on top of LN layer through plasma-enhanced chemical vapor deposition (PECVD). Then, with a 200 nm-thick electron beam resist (PMMA-A4) spin-coated on α-Si layer, the patterns of on-chip metasurface and grating couplers were defined by another EBL process and transferred to an electron beam evaporated chromium layer after development, which served as a hard mask for the following dry etching process of the α-Si layer in a mixture of C_4_F_8_ and SF_6_ plasma. The remaining chromium mask was removed by ammonium cerium nitrate. As a next step, two layers of LOR5B film and AZ5214 film were spin-coated and followed by an ultraviolet (UV) exposure to define the electrode patterns. After development, a 400 nm thick gold film was deposited onto the wafer and then a lift-off process transferred the patterns of electrodes. At last, the fabricated chip was mounted onto a printed circuit board (PCB) with wire-bonding which connected the electrodes of LN MZI switch to the PCB.

### Measurement setup

A 1550 nm laser was incident onto the grating coupler through an optical fiber with polarization controller to excite TE_0_ guided mode. The guided wave radiation extracted by on-chip metasurface was subsequently collected by an objective (40×, NA = 0.6) and performed Fourier transformation by a Fourier lens in front of a CCD camera to acquire far-field intensity distributions. In order to access dynamic modulation, a three-channel digital-to-analog converter was utilized to apply independent square wave voltages onto the electrodes of two-stage LN MZI switch. The transmission of each output port of LN MZI switch was obtained using an optical power meter when applying triangular wave voltages. The response speed of LN modulators was characterized through an avalanche photodiode connecting with an oscilloscope to record the instantaneous response of transmission upon the applied 10 kHz square wave voltage via an arbitrary waveform generator.

## Supplementary information

Suppementary Video

Supplementary information for Dynamic holographic display with addressable on-chip metasurface network based on lithium niobate photonics

## Data Availability

The source data are available from the corresponding author upon reasonable request. All data needed to evaluate the conclusion are present in the manuscript and/or the Supplementary Information.
