# Supplementary material for: Dynamic holographic display with addressable on-chip metasurface network based on lithium niobate photonics
Source: Light Sci Appl. 2025 Sep 18;14:332. doi: 10.1038/s41377-025-02014-6 (PMC12446423; doi:10.1038/s41377-025-02014-6)
Supplement: Supplementary file 2 — Supplementary information for Dynamic holographic display with addressable on-chip metasurface network based on lithium niobate photonics [file 41377_2025_2014_MOESM2_ESM.docx]

Supplementary Information for

**Dynamic holographic display with addressable on-chip metasurface network based on lithium niobate photonics**

Jitao Ji^†^, Zhilin Ye^†^, Zhizhang Wang^*^, Jiacheng Sun, Xueyun Li, Jian Li, Junyi Wang, Bin Fang, Zihan Gao, Shanshan Hu, Shining Zhu & Tao Li^*^

*National Laboratory of Solid State Microstructures, Key Laboratory of Intelligent Optical Sensing and Manipulations, Jiangsu Key Laboratory of Artificial Functional Materials, College of Engineering and Applied Sciences, Nanjing University, Nanjing, 210093, China.*

† These authors contributed equally to this work.

*Correspondence and requests for materials should be addressed to Zhizhang Wang, [zhizhangwang@nju.edu.cn](mailto:zhizhangwang@nju.edu.cn), and Tao Li, [taoli@nju.edu.cn](mailto:taoli@nju.edu.cn).

**Note 1. Comparison of the performance of existing metasurfaces**

To date, how to realize fast dynamic modulation along with sufficient information capacity in a compact system remains a critical challenge in the existing metasurfaces for practical applications. Table S1 lists the metrics of existing free-space and on-chip metasurfaces. For free-space metasurfaces whose dynamic control capabilities are typically actuated by MEMS, metallic polymer and flexible substrate^1-3^, they tend to suffer from relatively low response speed. With regard to on-chip metasurfaces, either there is a lack of dynamic modulation functionality^4-6^, or the number of modulation channels is limited^7-9^, which hinders their developments in practical applications. To this end, this work establishes an on-chip metasurface network (OCMN) framework to significantly enhance the number of modulation channels while providing an integrated and ultra-fast dynamic modulation strategy for metasurfaces by virtue of a lithium niobate (LN) electro-optical switch on LNOI platform. It should be noted that although the number of modulation channels demonstrated in this work is 4×4, such configuration is universal and scalable, which could be further expanded into a larger OCMN and compatible with other metasurface design strategies to both increase the modulation channels and improve the quality of guided wave radiation.

Table S1. The metrics and performance of the existing free-space and on-chip metasurfaces.

| **Ref.** | **Mechanism** | **Channels** | **Platform** | **Dynamic control** | **Speed** | **Potential application** | |
| --- | --- | --- | --- | --- | --- | --- | --- |
| [1] | Propagation | N.A. | free space | MEMS | ~ms | Imaging | |
| [2] | Geometric | 2 | free space | metallic polymer | 20.8 ms | AR/VR | |
| [3] | Geometric | 3 | free space | Flexible substrate | N.A. | Display | |
| [4] | Resonant | 1 | silicon nitride | N.A. | N.A. | Display/Communication | |
| [5] | Geometric | 6 | silica | N.A. | N.A. | AR display | |
| [6] | QBIC | 4 | polymer | N.A. | N.A. | AR/LiDAR | |
| [7] | Geometric,  Detour | 4 | silicon nitride | Liquid crystal polarizer | ~ms | Display | |
| [8] | Detour | 2 | hydrogel waveguide | Humidity | 310 ms | AR device | |
| [9] | Geometric, Propagation | 2 | lithium niobate | LN EO modulator | ~ns | Communication | |
| This work | Geometric,  Detour | 4×4^‡^ | lithium niobate | LN EO switch | ~6.5 ns | | Display/optical interconnects |

‡ Scalable on-chip metasurface network

**Note 2. Diatomic arrangement based on geometric phase and detour phase**


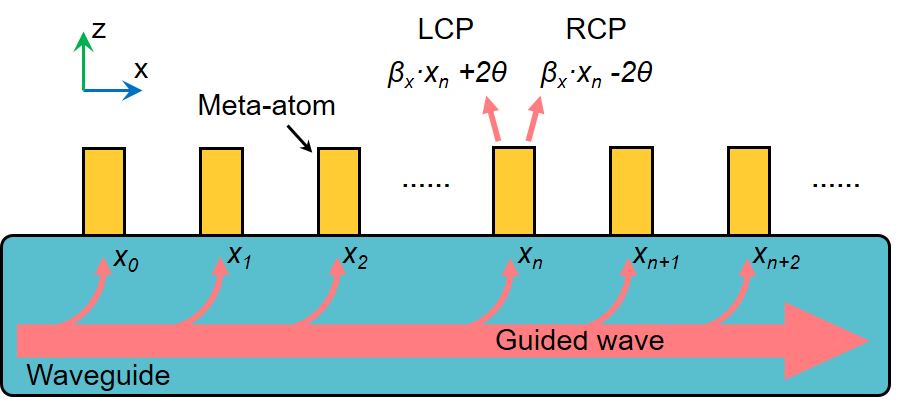


Fig. S1 The joint modulation mechanism of geometric phase and detour phase for guided wave radiation.

As demonstrated in Fig. S1, the process of guided wave radiation modulated by on-chip metasurface can be regarded as the following steps. For simplicity, we take the guided wave propagating along the x direction for example. First, the input TE_0_ guided wave would accumulate detour phase of $\beta_{x}\cdot x_{n}$ during propagation once encountering the nth meta-atom with an x-coordinate of $x_{n}=n\cdot P_{x}+\delta_{x}$. Subsequently, modulated by the meta-atom with a rotation angle of *θ*, the guided wave which can be considered as a linearly polarized mode would be extracted into left circularly polarized (LCP) and right circularly polarized (RCP) parts with geometric phase of 2*θ* and -2*θ*. The phase of the extracted LCP and RCP guided wave radiation and by each meta-atom can thereby be written as

(S1)

Because of the global periodical arrangement of meta-atoms with period of *P_x_* = λ*_effx_* along x direction, Eq. (S1) could be simplified as

(S2)

It should be noted that the polarization conversion efficiency of on-chip metasurface based on geometric phase is not sufficiently high, thus there remains unmodulated part of guided wave radiation with a phase of , which only experience detour phase but is not modulated by geometric phase (as shown in Fig. S2a). This phenomenon could result in unwanted background patterns and decrease the signal-to-noise ratio (SNR) of the generated far-field image. To illustrate this effect, we conduct numerical simulations of monatomic on-chip metasurface for comparison. Figure S2b lists the corresponding generated holograms under TE_0_ mode illumination along x direction, indicating guided wave radiation with low SNR (defined as the ratio of the intensity of the target pattern to that of the total pattern in the far field) both for LCP and RCP images. To address the above issue, diatomic structure consisting of nanopillars A and B depicted in Fig. S2c is employed to avoid the unmodulated part of guided wave radiation. With x and y displacements of *P_x_*/2 and *P_y_*/2, the phase difference between the unmodulated radiations of nanopillars A and B turns out to be π, leading to a destructive interference and therefore elimination of background noise. Meanwhile, the modulated LCP and RCP radiation of nanopillars A and B by detour phase and geometric phase acquires the phase modulation as follows.

(S3)

Due to the orthogonal arrangement of two nanopillars, the modulated radiations of nanopillars A and B preseent the same phase profiles (i.e., and ), which contributes to constructive interference for high SNR guided wave radiations shown in Fig. S2d. Therefore, the phase responses of such diatomic structure could be described in accordance with Eq. (S2) without any unmodulated components. With regard to the guided wave propagating along the y direction, the phase profile of the guided wave radiation based on diatomic structure could be similarly derived as

(S4)


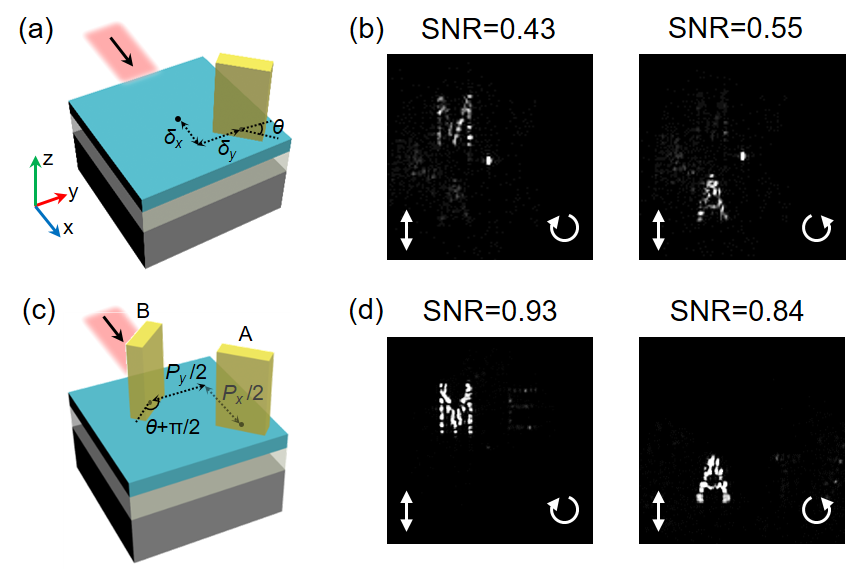


Fig. S2 Comparison of the signal-to-noise ratio of holograms generated by monatomic and diatomic on-chip metasurfaces. (a) Schematics of monatomic structure and (b) the corresponding holograms under TE_0_ mode illumination along x direction. (c) Illustration of diatomic structure and (d) the generated holograms with high signal-to-noise ratio.

**Note 3. Modified G-S algorithm for phase distributions of four multiplexed channels**


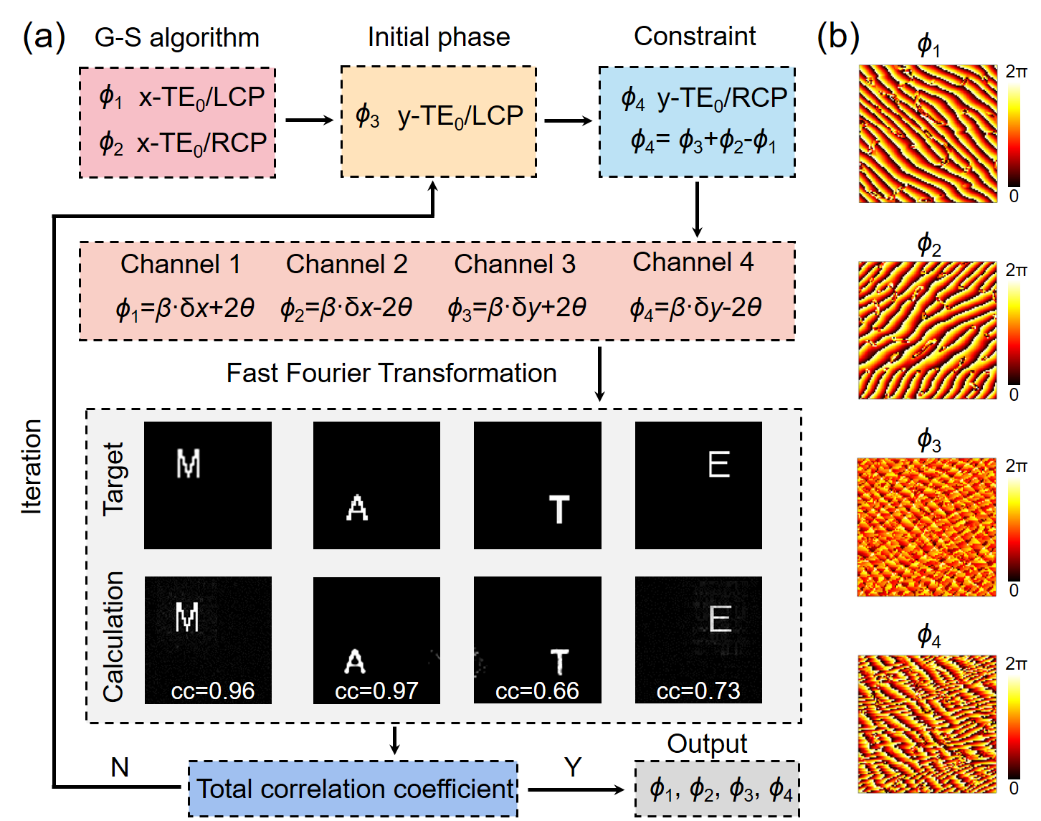


Fig. S3 Design process of on-chip four-channel meta-holography. (a) Flowchart of the optimization process to obtain the four phase maps with three design variables. cc: correlation coefficient. (b) Optimized phase distributions of four channels with 100×100 pixels.

With three independent design variables, we attempt to encode four different holographic images into a single on-chip metasurface based on geometric phase and detour phase. To determine the phase maps of four channels with the constraint of and minimize the reconstructed deviation from the target images, an iterative calculation process is adopted as illustrated in Fig. S3a. As a first step, two phase distributions *ϕ*_1_ and *ϕ*_2_ of channel 1 and channel 2 for TE_0_ guided wave illumination along x direction are obtained through the Gerchberg–Saxton (G-S) algorithm and serve as initial conditions for optimization of *ϕ*_3_ and *ϕ*_4_ for guided wave illumination along y direction. Then the phase profile *ϕ*_3_ of channel 3 is first endowed with random phase and the relation of as a constraint is used to connect channel 4 with other three channels. As such, the phase distributions of four channels are obtained and the corresponding four holographic images can be retrieved through fast Fourier transformation (FFT). Meanwhile, the total correlation coefficient of the generated holographic images by channel 3 and channel 4 is utilized as evaluation function to assess the quality of reconstructed images. The phase distribution *ϕ*_3_ is subsequently optimized through a feedback function and acts as the initial condition for next loop iteration. As a consequence, *ϕ*_3_ and *ϕ*_4_ are updated in every iteration step until satisfying phase distributions are found for high-quality holography. Eventually, in virtue of the optimization algorithm, four holographic letters “M”, “A”, “T” and “E” are achieved in the far-field zone with three independent design variables and the corresponding phase maps of four channels are exhibited in Fig. S3b.

**Note 4. Phase compensation for bended wavefront of guided waves**


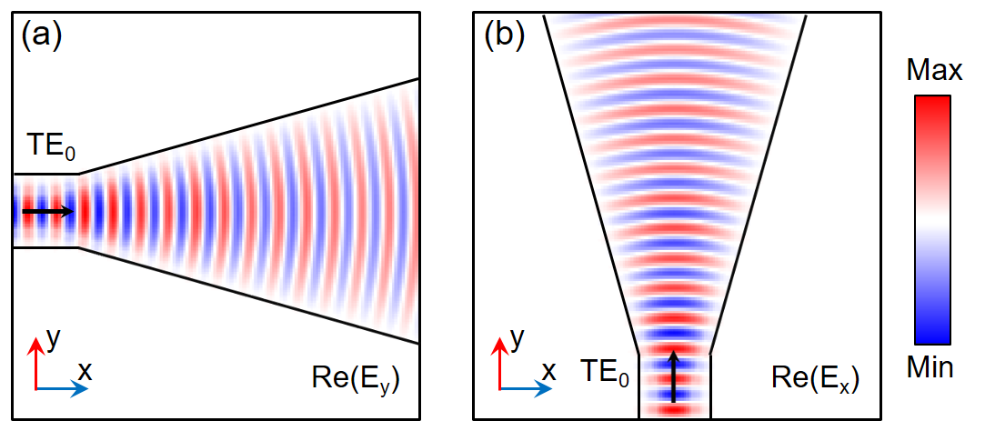


Fig. S4 Phase correction for bended wavefront of guided waves. The partial electric field profiles of guided wave illumination from a single-mode LN waveguide to a tapered adiabatic waveguide along (a) x and (b) y directions.

To illuminate on-chip metasurfaces with TE_0_ modes propagating from a single-mode waveguide to a slab waveguide, an adiabatic taper is utilized to connect the grating coupler to the slab waveguide region. As a result, the incident TE_0_ mode presents bent wavefront during the propagating process, which should be considered as the incident phase profile for the subsequent phase modulation by on-chip metasurface. To acquire the contour of the bent wavefront, we employ numerical simulations for TE_0_ guided wave illumination along x and y directions in LN waveguides with an etch depth of 300 nm from a 1 μm-width single-mode waveguide to a 100 μm-width slab waveguide. Figure S4 shows the partial electric field profiles of the TE_0_ guided wave and the bended wavefront could thereby be obtained as *ϕ_bx_* and *ϕ_by_* for guided wave illuminations along x and y directions, respectively. Hence, the targeted phase distributions of four multiplexed channels are corrected to be

(S5)

where *ϕ*_1_, *ϕ*_2_, *ϕ*_3_ and *ϕ*_4_ are the phase distributions of four channels optimized through the modified G-S algorithm described in Fig. S3.

**Note 5. Evaluation of crosstalk between four multiplexed channels**

In order to quantify the crosstalk between four multiplexed channels of on-chip metasurface, the correlation coefficient is introduced as a quantitative metric. As displayed in Fig. S5, the correlation matrix of the experimentally measured four-channel holographic images shown in Fig. 2f and the target images indicate that the diagonal terms are all above 0.8 while all the non-diagonal terms are below 0.05. Such quantitative results confirm that the reconstructed four multiplexed holographic images are of high quality and almost negligible crosstalk.


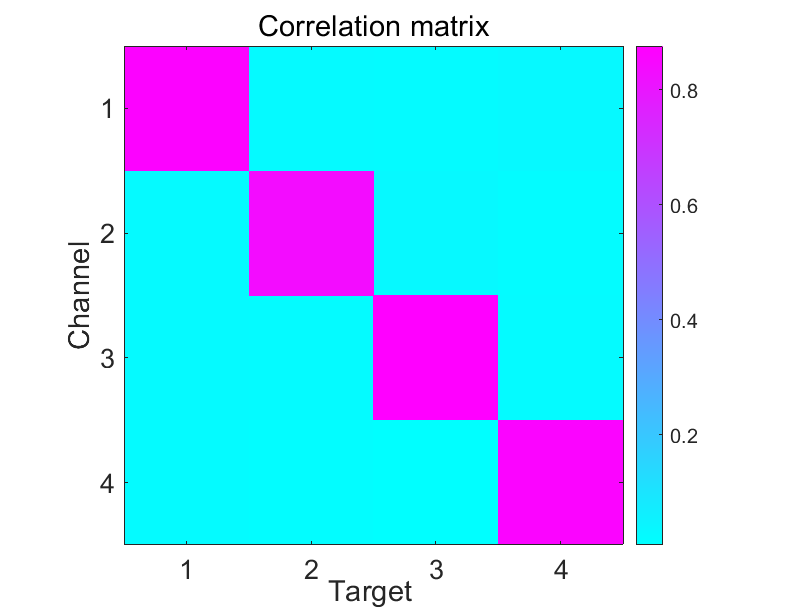


Fig. S5 Correlation coefficients matrix of the measured holographic images in Fig. 2f and targeted images.

**Note 6. Broadband response of on-chip metasurface**


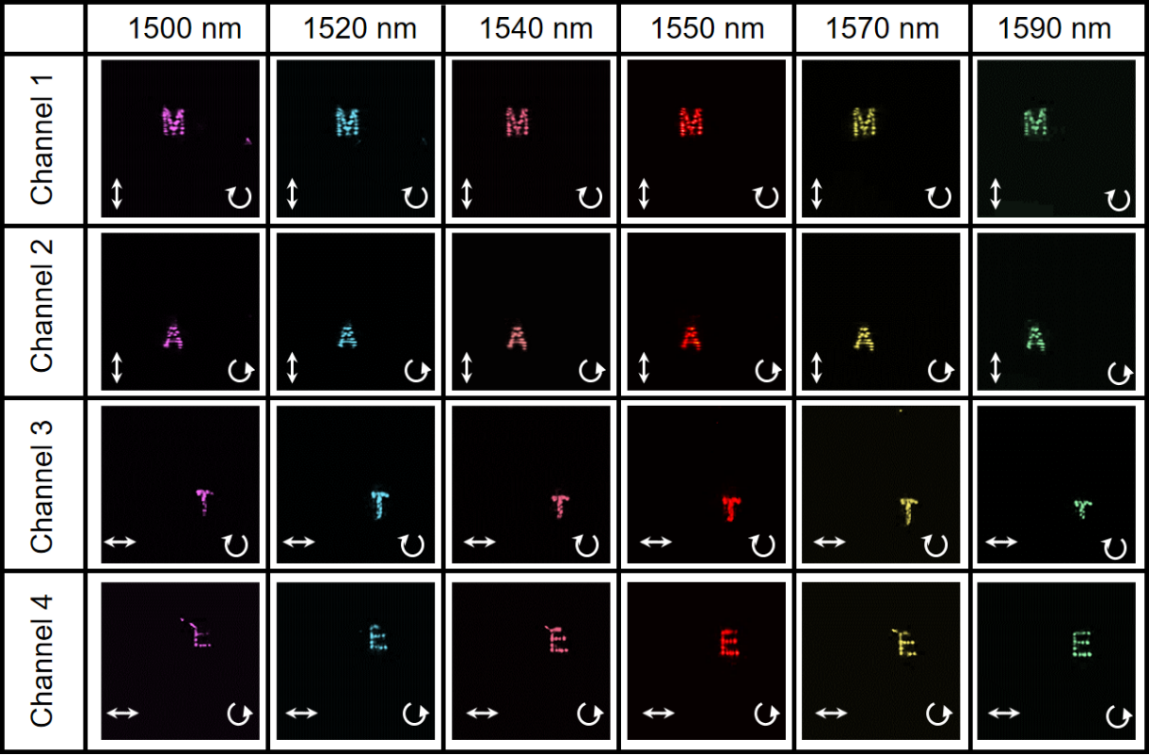


Fig. S6 Broadband properties of the proposed on-chip metasurface based on geometric phase and detour phase from 1500 nm to 1590 nm with false colors.

Due to the broadband response of geometric phase and detour phase, the designed on-chip metasurface demonstrates multiplexed guided wave radiation capability over a wide spectral range. For the incident guided waves with an off-center wavelength *λ*, the phase responses could be rewritten as follows according to Eqs. (S1) and (S2)

(S6)

Here, *β_x_*(*λ*) and *β_y_*(*λ*) are the propagation constants of TE_0_ modes propagating along x and y directions at wavelength of *λ*. Since the meta-atoms are in a global periodic arrangement of *P_x_* = λ*_effx_* and *P_y_* = λ*_effy_* with displacements of *δ_x_* and *δ_y_*, the above phase responses could be derived as

(S7)

where Δ*β_x_*(*λ*) and Δ*β_y_*(*λ*) represent the difference between the effective wavelengths of TE_0_ modes at wavelength *λ* and central wavelength of 1550 nm along x and y directions, respectively. It is observed that additional phase gradient distributions (Δ*β_x_*(*λ*)·*n*·*P_x_* and Δ*β_y_*(*λ*)·*n*·*P_y_*) are induced in Eq. (S7) compared with Eq. (1), which would cause deflection of guided wave radiation at off-center wavelengths. Nevertheless, the latter components in Eq. (S7) still satisfy the targeted phase profiles and would take effect for generating four-channel multiplexed holographic images. In experiments, as displayed in Fig. S6, the measured holographic images in four channels exhibit maintained imaging quality with negligible degeneration over a broadband spectrum ranging from 1500 nm to 1590 nm. Typically, the holographic letters “M” and “A” slightly deflect to the left side and holographic letters “T” and “E” gradually go downwards as the wavelengths increase, whereas the additional deflection angles pose no impact on the quality of holographic images. The above results illustrate the broadband performance of the proposed on-chip metasurface based on geometric phase and detour phase in terms of multiplexed guided wave radiation.

**Note 7. Demonstration of multiplexed orbital angular momentum beams**

In addition to holographic images, we also design on-chip multiplexed metasurface for generation of orbital angular momentum (OAM) beams with regard to the potential applications such as OAM optical communications and optical tweezers. Here, we design the targeted phase distribution of four multiplexed channels as

$\begin{matrix} \phi_{xL}=l_{1}\varphi\text{，}\phi_{xR}=l_{2}\varphi\\ \phi_{yL}=l_{3}\varphi\text{，}\phi_{yR}=l_{4}\varphi\end{matrix}$ (S8)

where *l*_1_=0, *l*_2_=1, *l*_3_=1 and *l*_4_=2 are the designed topological charges while *φ* represents the azimuth angle. Figure S7a shows the fabricated on-chip metasurface based on geometric phase and detour phase on top of a LN waveguide. Once illuminated by 1550 nm TE_0_ guided modes, the on-chip metasurface would extract the propagating guided wave and mold it into free space in the form of diverse OAM beams, as listed in Fig. S7b. Through switching the illumination direction and rotating the direction of polarization analyzers, OAM beams with topological charges of *l*_1_=0, *l*_2_=1, *l*_3_=1 and *l*_4_=2 could be achieved as desired.


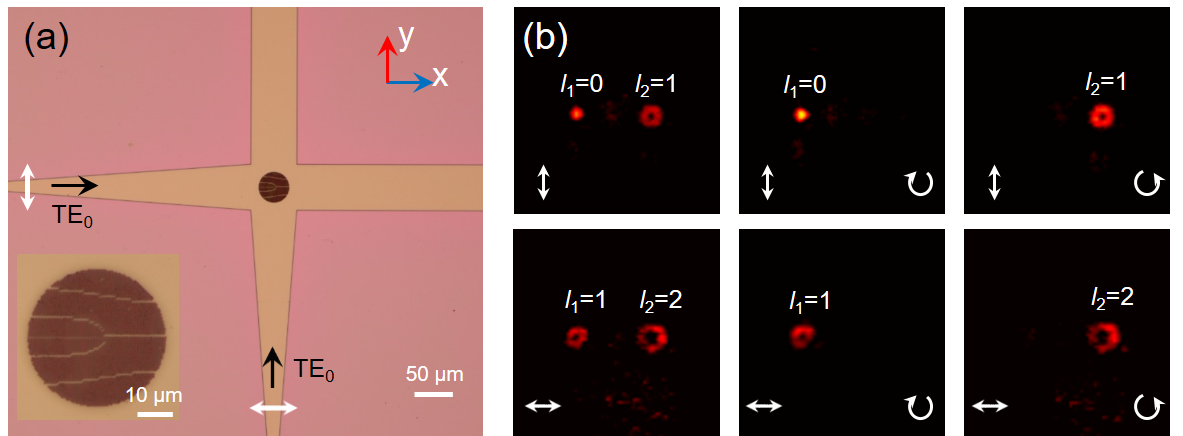


Fig. S7 Experimental demonstration of generating orbital angular momentum beams. (a) Photograph of the fabricated on-chip metasurface on top of a LN waveguide crossing. Inset: the enlarged image of the on-chip metasurface. (b) The generated far-field intensities under guided wave illuminations along x and y directions without/with LCP and RCP analyzers.

**Note 8. Designed holographic patterns of four on-chip metasurfaces in OCMN**

Table S2 lists the designed holographic letters and numbers of four multiplexed channels of each on-chip metasurface MS 1-4 in OCMN shown in Fig. 3. As an illustration, when guided waves are excited in Port 1, they will propagate along the x direction and are subsequently radiated by MS 1 and MS 2 into free space. According to Table S2, MS 1 and MS 2 would project far-field holographic patterns of “9” and “E” (“7” and “h”) under LCP (RCP) analyzer respectively, therefore contributing to a combined pattern of “9E” (“7h”) as displayed in Fig. 3c-d. By analogy, by switching the input port from Ports 2 to 4, the holographic patterns would be presented as “54”, “n3” and “qp” (“26”, “db” and “LU”) after passing through LCP (RCP) analyzer, respectively.

Table S2. The designed holographic patterns of MS 1-4 in OCMN.

| **Metasurface/Channels** | ***ϕ_xL_*** | ***ϕ_xR_*** | ***ϕ_yL_*** | ***ϕ_yR_*** |
| --- | --- | --- | --- | --- |
| MS 1 | 9 | 7 | 3 | d |
| MS 2 | E | h | q | U |
| MS 3 | 4 | 2 | n | b |
| MS 4 | 5 | 6 | p | L |

**Note 9. Design of lithium niobate devices**

Note 9a. Optimization of multi-mode interference couplers

In the two-stage LN MZI switch, each LN modulator consists of a 1×2 multi-mode interference (MMI) coupler, a pair of LN phase modulators in two arms and a 2×2 MMI coupler. Therefore, to establish high-performance LN modulators, passive LN devices including 1×2 and 2×2 MMI couplers require elaborate design to achieve high transmission and a high extinction ratio. Using particle swarm optimization, we optimize the width and length of 1×2 MMI coupler to be 5.1 μm and 18.1 μm, respectively, which demonstrates a transmission of 48.5% at 1550 nm as shown in Fig. S8a. The inset in Fig. S8a depicts the related optical field distribution at 1550 nm. Based on the similar optimization process, the transmission at each output port of 2×2 MMI coupler is designed to be 49.2% with an optimized width of 8.5 μm and length of 65.9 μm, as plotted in Fig. S8b. To characterize the extinction ratio of the designed 2×2 MMI coupler, the phase difference between two input ports I_1_ and I_2_ is set to be 90°. Figure S8c presents the transmission of two output ports O_1_ and O_2_, indicating a high extinction ratio of 28.2 dB at 1550 nm.


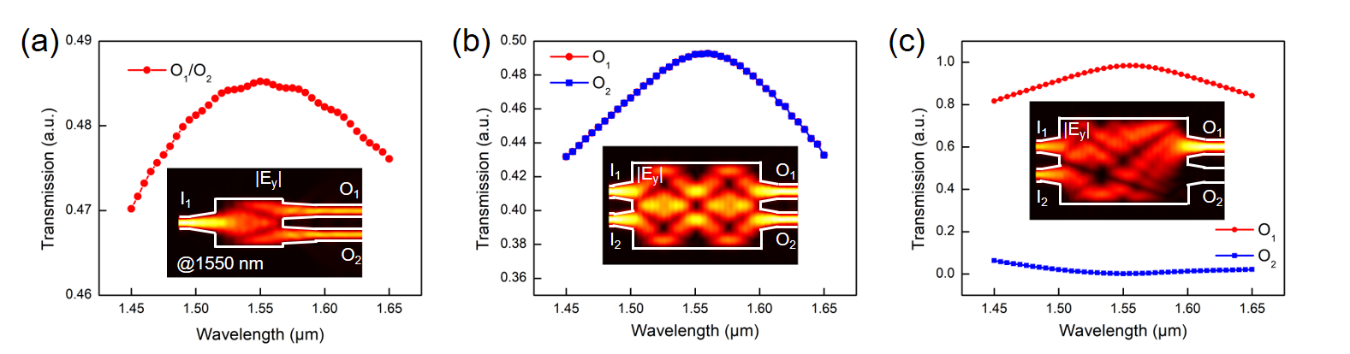


Fig. S8 Design of LN MMI couplers. (a) The transmission of two output ports of 1×2 MMI coupler. Inset: The optical field distribution in 1×2 MMI coupler at 1550 nm. The transmission of two output ports of 2×2 MMI coupler with phase difference of (b) 0 and (c) π/2 between two input ports. Insets: The corresponding optical field distributions at 1550 nm.

In experiments, six cascaded MMI couplers with seven output ports (O_1_-O_7_) were fabricated to estimate the insertion loss, as shown in Fig. S9a. The insertion loss of MMI coupler can be measured by the power attenuation at each port, as plotted in Fig. S9b. Through recording the power of output ports at each branch, the slope of power attenuation could be linearly fitting to -3.48 dB, which indicates an insertion loss of 0.48 dB for each MMI coupler considering the slope would be -3 dB if the insertion loss is zero.


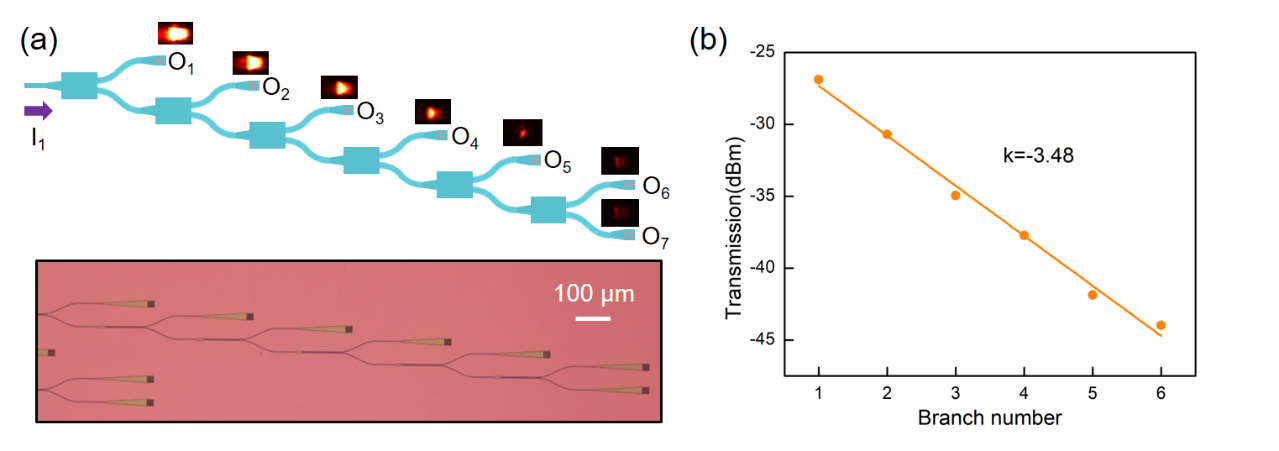


Fig. S9 Evaluation of insertion loss of MMI coupler. (a) Cascaded six MMI coupler with seven output ports. (b) The transmission power of six output ports at each branch.

Note 9b. Design of lithium niobate modulators

With regard to active LN integrated devices, the design of LN modulators should take into consideration the aspect of both modulation efficiency and optical loss. The schematics of LN modulators are plotted in Fig. S10a and S10b with design variables of electrodes gap g and etch depth h_ridge_. The modulation efficiency could be evaluated by half-wave voltage length product V_π_·L, which can be derived by the following steps. First, the electric field distributions E_y_ around the LN waveguide can be obtained through numerical simulations when applying a bias voltage of V_b_ onto source and ground electrodes, as displayed in Fig. S10c with the simulated mode profile of fundamental TE mode at 1550 nm. With such electric field distributions, the change of refractive index Δ*n* of LN material could be subsequently calculated as

$\Delta n=\frac{E_{y}n_{e}^{3}\gamma_{33}}{2}$ (S9)

Here, *n_e_* and *γ*_33_ are the extraordinary refractive index and electro-optic coefficient of x-cut thin-film LN material. According to Δ*n* in LN material, the change in effective refractive index of TE_0_ mode Δ*n_neff_* in LN waveguide can thus be acquired through simulations. Then, in view of the push-pull configuration adopted in our LN modulator design, the length L of the modulation region should satisfy $L=\lambda/4\Delta n_{eff}$ to induce phase difference of π between two arms of modulators. Therefore, V_π_·L can be obtained by the product of applied bias voltage V_b_ and required modulation length L. Figure S10d lists the value of V_π_·L as a function of electrodes gap g and etch depth h_ridge_. It reveals that a smaller gap and a lower etch depth increase the electro-optical overlap integral and contribute to higher modulation efficiency. However, as a trade-off, small electrode gap and low etch depth will lead to large optical loss in the metal electrode and a weak guided mode confinement, respectively. To make a balance between modulation efficiency and optical loss, we select g and h_ridge_ to be 6.5 μm and 300 nm, contributing to a relatively low V_π_·L of 2.8 V·cm. Based on the above design, we fabricate the LN electro-optic modulator shown in Fig. S10e and analyze its modulation efficiency by measuring the transmission of output port when applying a triangular wave voltage onto source and ground electrodes. As plotted in Fig. S10f, the transmission presents a sinusoidal relationship with applied voltages, indicating V_π_·L of 4.2 V·cm and extinction ratio (ER) of 10.35 dB. Compared with simulation results, the relatively higher half-wave voltage length product and lower ER measured in experiments might be attributed to the fabrication imperfection.

In general, in addition to modulation efficiency and optical loss, the physical size of travelling wave electrodes is another factor that requires attention in the design of modulators to reach impedance matching and phase velocity matching conditions for large modulation bandwidth. While in this work, to achieve addressable guided wave radiation, we only concentrate on the optimization of modulation efficiency without considering modulation bandwidth. Nevertheless, the response time of the designed LN modulator still reaches on the order of nanoseconds, demonstrating an ultra-fast addressing capability. Further optimization on travelling wave electrodes could be carried out to improve the modulation bandwidth of the modulator.

With regard to the power consumption of the whole device, it is mainly determined by the LN electro-optical switch composed of three LN modulators for addressing operation. Due to Pockels EO effect, the power consumption of LN modulator could be described by energy change^10-11^. The source and ground electrodes in LN modulator can be regarded as a capacitor and its capacitance is measured to be 39 pF in experiments. By taking it into consideration that the measured half-wave voltage is 4.2 V, the power consumption of each modulator could be estimated to be 343 pJ, which contributes to a total power consumption of ~1.03 nJ per switching operation for the whole device.


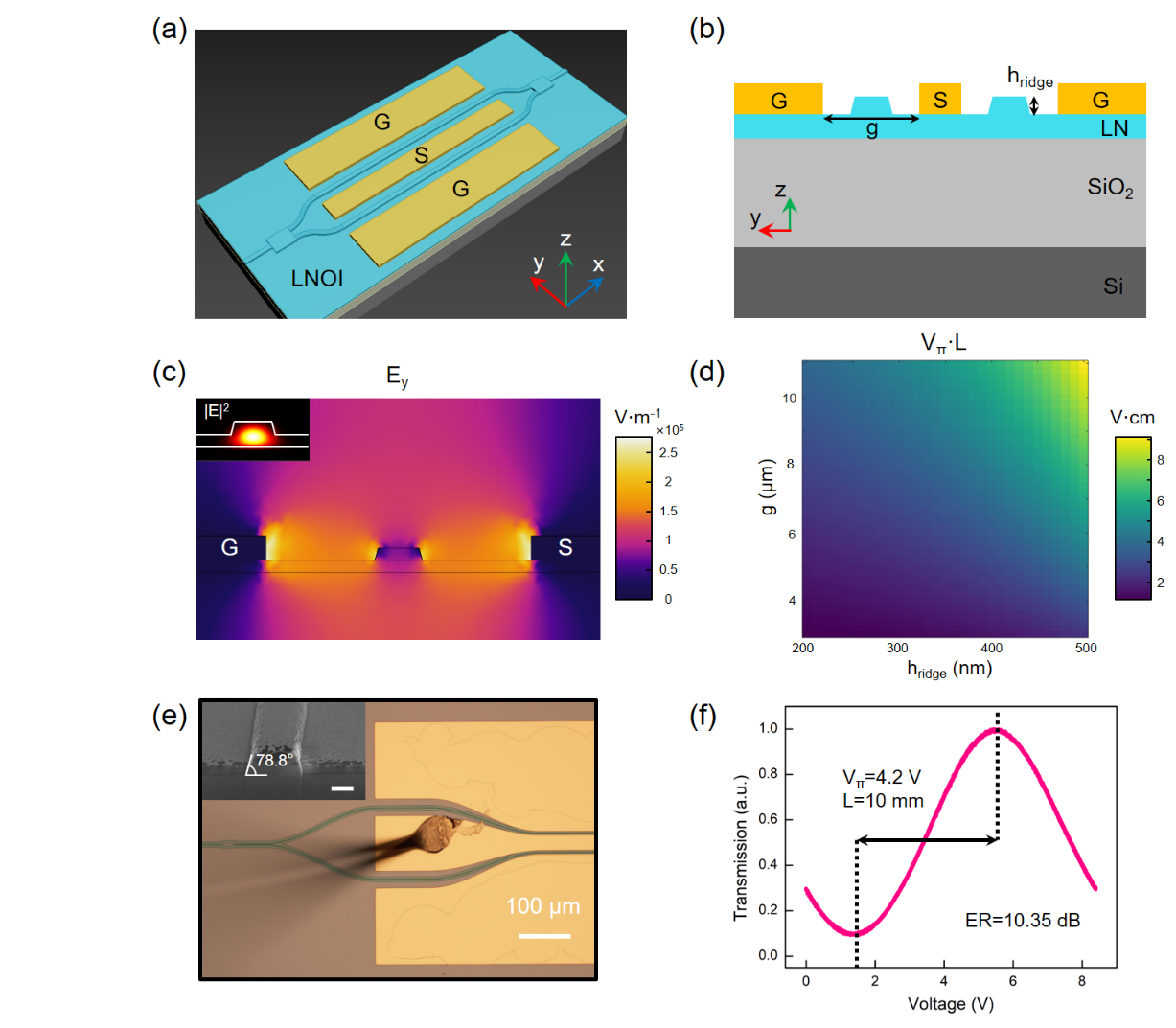


Fig. S10 Design of LN electro-optic modulators. (a) Conceptual diagram and (b) side view of the designed LN modulator. (c) The simulated electric field distribution E_y_ around the source and ground electrodes with a bias voltage of 1 V. Inset: The mode profile of TE_0_ mode. (d) The half-wave voltage length product as a function of the ridge height h_ridge_ of LN waveguide and the gap g between source and ground electrodes. (e) The optical microscope image of the prepared LN modulator. Inset: Side view of scanning electron microscope image of the fabricated LN waveguide. Scale bar: 0.5 μm. (f) The measured transmission of the output of LN modulator when applying a triangular wave voltage.

**References**

1. Arbabin, E. et al. MEMS-tunable dielectric metasurface lens. *Nature Communications* **9**, 812 (2018).

2. Karst, J. et al. Electrically switchable metallic polymer nanoantennas. *Science* **374**, 612-616 (2021).

3. Wang, Z. Z. et al. Gesture-interactive dynamic holo-display via topography flexible metasurfaces. *ACS Nano* **19**, 1286-1294 (2025).

4. Guo, X. X. et al. Molding free-space light with guided wave-driven metasurfaces. *Science Advances* **6**, eabb4142 (2020).

5. Liu, Z. Y. et al. Broadband spin and angle co-multiplexed waveguide-based metasurface for six-channel crosstalk-free holographic projection. *eLight* **4**, 7 (2024).

6. Huang, H. Q. et al. Leaky-wave metasurfaces for integrated photonics. *Nature Nanotechnology* **18**, 580-588 (2023).

7. Shi, Y. Y. et al. Electrical-driven dynamic augmented reality by on-chip vectorial meta-display. *ACS Photonics* **11**, 2123-2130 (2024).

8. Li, X. L. et al. Hydrogel‐waveguiding on‐chip meta‐optics for dynamic multicolor holography. *Advanced Functional Materials* **34**, 2408958 (2024).

9. Zhong, H. Z. et al. Gigahertz-rate-switchable wavefront shaping through integration of metasurfaces with photonic integrated circuit. *Advanced Photonics* **6**, 016005 (2024).

10. Yue, G. C. et al. Integrated lithium niobate optical phased array for two-dimensional beam steering. *Optics Letters* **48**, 3633-3636 (2023).

11. Wang, Z. Z. et al. Fast-speed and low-power-consumption optical phased array based on lithium niobate waveguides. *Nanophotonics* **13**, 2429 (2024).
